# Comparison of SARS-CoV-2 molecular results from the first two COVID-19 waves in Gauteng

**DOI:** 10.4102/sajid.v39i1.647

**Published:** 2024-11-22

**Authors:** Kreshalen Govender, Rendani T. Mafuyeka, Azwidowi Lukhwareni, Pieter Meyer

**Affiliations:** 1 Department of Medical Virology, Faculty of Health Sciences, University of Pretoria, Pretoria, South Africa; 2 Department of Virology, Tshwane Academic Division, National Health Laboratory Service, Pretoria, South Africa; 3 Department of Molecular Virology, Faculty of Virology, Ampath Laboratories Inc., Pretoria, South Africa; 4 Department of Immunology, Faculty of Health Sciences, University of Pretoria, Pretoria, South Africa; 5 Department of Immunology, Tshwane Academic Division, National Health Laboratory Service, Pretoria, South Africa

**Keywords:** SARS-CoV-2, RT‒PCR, cycle threshold, demographics, COVID‒19

## Abstract

**Background:**

Laboratory-based molecular assays return cycle threshold (Ct) values for each gene target. There is limited hyperlocal information describing the Ct, age and sex trends during the severe acute respiratory syndrome coronavirus-2 (SARS-CoV-2) waves in South Africa.

**Objectives:**

To analyse the demographic and Ct value trends of SARS-CoV-2 molecular assays from two South African hospitals.

**Method:**

The Seegene Allplex 2019-nCoV™ results from the first two waves (June–July 2020 and November 2020–January 2021) from two major hospitals in Gauteng, South Africa, were extracted from the laboratory information system. Demographic variables and Ct values were analysed.

**Results:**

Overall, 2391 samples were analysed over two waves. In both waves, more women were tested than men; 68.4% versus 31.2% in the first wave and 59.8% versus 39.7% in the second wave. Differences in Ct values among the age groups were non-significant overall; however, most median Ct values in all age groups were < 30. Men had lower median Ct values in the first wave, but this trend reversed in the second wave (*p* < 0.001). The first wave had significantly lower mean and median Ct values per gene target (*p* < 0.001).

**Conclusion:**

Patients tested in the first wave had lower Ct values. All age groups in both waves demonstrated infectivity potential; the demographic analysis agreed with South Africa’s coronavirus disease 2019 (COVID-19) epidemiological trends in both waves.

**Contribution:**

Granular insight into the basic demographic variables and Ct trends of SARS-CoV-2 real-time polymerase chain reaction (RT-PCR) results within and between SARS-CoV-2 waves in South Africa.

## Introduction

Laboratory-based real-time polymerase chain reaction (RT‒PCR) remains the gold standard for detecting severe acute respiratory syndrome coronavirus-2 (SARS-CoV-2).^1^ Most qualitative kits used in the public health sector, approved by the United States Food and Drug Administration and the South African Health Products Regulatory Authority, target multiple conserved regions on the viral genome and report a cycle threshold (Ct) value per detected gene.^2,3,4^

Commonly targeted SARS-CoV-2 genes include the envelope (*E*), nucleocapsid (*N*) and ribonucleic acid-dependent ribonucleic acid polymerase (*RdRp*) genes, enabling reliable detection of archetype viruses and variants.^5^ Conventionally, Ct values are considered a proxy for viral titre and infectivity potential. However, various pre-analytical and analytical factors may affect the generated Ct value.^6^

South Africa’s coronavirus disease 2019 (COVID-19) pandemic consisted of five waves. The country’s molecular diagnostic infrastructure, established for its human immunodeficiency virus (HIV) management programme, enabled rapid upscaling of molecular testing for SARS-CoV-2 using high-performance assays. Within National Health Laboratory (NHLS) centres conducting testing, the most utilised kit was the Seegene Allplex™ 2019-nCoV, which targets the three SARS-CoV-2 genes (*E, RdRp* and *N*) and has acceptable performance in detecting viral variants.^7^

Notably, each wave was driven by unique variants or sub-variants, which affects epidemiological trends and diagnostic assays.^8,9^ The impacts on diagnostic tests included gene target failure, delayed amplification and false negative results; the last being less likely owing to the multitarget design of kits from major manufacturers. Moreover, the high rates of mutation and resulting genomic plasticity ensure viral circulation within a population and adversely affect the performance of diagnostic platforms.^10^

The first wave of the COVID-19 pandemic was dominated by the ancestral strain with a D614G mutation, while the second wave was driven by the beta (B.1.351) variant.^11^ International studies describing epidemiological or Ct value trends within or between waves are variable, with some authors describing significant differences and others reporting negligible differences.^12,13,14^ There are few studies at facility level describing Ct value trends of the beta variant after its emergence in South Africa towards the latter half of 2020.

Pre-analytical factors also impact the performance of molecular platforms; the unprecedented burden of disease seen in the second wave led to overwhelming sample volumes at diagnostic laboratories worldwide resulting in inappropriate sample storage and delayed testing.^15^

Testing practices remained iterative throughout the pandemic; early guidelines published by the National Institute for Communicable Diseases (NICD) had a low threshold of testing eligibility leading to high sample volumes from inpatients and outpatients in both waves.^16,17^

Comparisons of Ct values, age and sex during South Africa’s SARS-CoV-2 waves are limited and analysis of past waves may provide further understanding of SARS-CoV-2’s evolution and subsequent public health implications.

This study also provides insight into the trends of SARS-CoV-2 molecular assay results at different timepoints and the epidemiological characteristics of samples tested early in the South African COVID-19 pandemic. Therefore, this study aimed to analyse the basic demographic variables and Ct values of patients during the first two waves from two hospitals in Gauteng, South Africa.

## Research methods and design

### Study design and setting

A retrospective comparative study was performed by obtaining SARS-CoV-2 RT‒PCR results from the Tshwane Academic Division (NHLS TAD) laboratory information system (LIS), TrakCare® (InterSystems, Massachusetts, United States of America).

The sex, age, *E* gene Ct, *RdRp* gene Ct and *N* gene Ct from the first two waves (01 June and 31 July 2020 [first wave]; 01 November 2020 and 31 January 2021 [second wave]) were analysed. These results were obtained by processing routine diagnostic samples from two major academic hospitals serving as major COVID-19 testing and care centres for inpatients in Pretoria, Gauteng, South Africa.

### Inclusion criteria

Severe acute respiratory syndrome coronavirus-2 positive RT-PCR test results from samples that were analysed using the Seegene Allplex™ 2019-nCOV (Seegene Inc., Korea) assay kit. Positive samples with Ct values of any of the three target genes, including patient age and sex, were obtained.

### Exclusion criteria

Data generated by any other kit were excluded. Invalid, inconclusive, negative, incomplete (absent Ct values or sex and age information) or duplicate results were excluded.

### Laboratory analysis

Respiratory samples in the form of swabs (oropharyngeal or nasopharyngeal) and aspirates (nasopharyngeal, tracheal, bronchial) were processed according to the laboratory’s standard operating procedure. Nucleic acid extraction was performed on either the Seegene Nimbus™ (Seegene Inc., Korea) or Roche MagnaPure™ (Roche Inc., California, United States of America) systems. Amplification and detection were performed on the Bio-Rad CFX™ (Bio-Rad, California, United States of America) platform using the Seegene Allplex™ 2019-nCoV kit, a real-time reverse transcription multiplex PCR assay targeting three regions of the SARS-CoV-2 genome (*E, RdRp, N*). A Ct value was generated for each detected target, with a positive result defined by NHLS TAD as any gene target with a Ct value < 40. The results were released to the LIS after quality control checks were performed.

### Data analysis

Data sorting, clean up and statistical analysis were performed using STATA 18.0 SE (StataCorp LLC, Texas, US). The non-parametric Shapiro‒Wilk test was used to analyse the data. The median and interquartile range (IQR) for numerical data are reported for patients tested per age group.

Categorical data were analysed using Pearson’s Chi-square test. Trends in Ct values and age groups within and between waves were performed using Kruskal‒Wallis tests and Dunn’s pairwise tests with Bonferroni correction. A *p*-value < 0.05 was considered significant.

### Ethical considerations

The study was approved by the University of Pretoria Research Ethics Committee of the Faculty of Health Sciences (approval number: 474/2021) and the NHLS Academic Affairs and Research Office (approval number PR2119150). The requirement for informed consent was waived as this was a retrospective, comparative, laboratory-based study without patient interaction. Patient confidentiality and data privacy were maintained in accordance with the Declaration of Helsinki and the *Protection of Personal Information Act, 2013 (*POPIA*)*.

## Results

**FIGURE 1:**
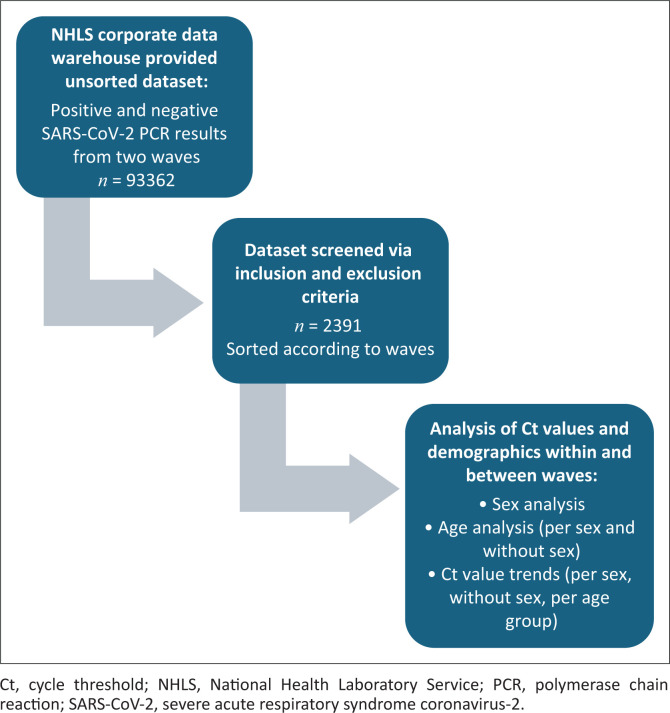
Flow chart depicting the derivation of the samples analysed.

A total of 93 362 SARS-CoV-2 PCR positive results from the first and second waves were initially extracted from the LIS. After removing duplicate results and applying inclusion and exclusion criteria, 2391 results were included in the final analysis, with 1252 (52%) from the first wave and 1139 (48%) from the second (Figure 1).

### Sex analysis

In both waves, the proportion of female to male samples was significantly greater (Pearson Chi^2^ = 18.92, *p* < 0.001). Overall, women were younger than men with a 5–6 years difference in median age (see Table 1).

**TABLE 1:** Sex and age analysis of severe acute respiratory syndrome coronavirus-2 real-time polymerase chain reaction patient results from two major academic hospitals in Gauteng per wave (June 2020 to July 2020 and November 2020 to January 2021).

Variables	*n*	%	Median age (years)	IQR	Min–Max
**Women**					
Wave 1	856	68	38	30–51	0–92
Wave 2	681	60	43	32–58	0–91
**Men**					
Wave 1	391	31	41	31–53	0–92
Wave 2	452	40	47	34–60	0–87

IQR, interquartile range; Min, minimum; Max, maximum; *n*, sample size.

### Age analysis

**FIGURE 2:**
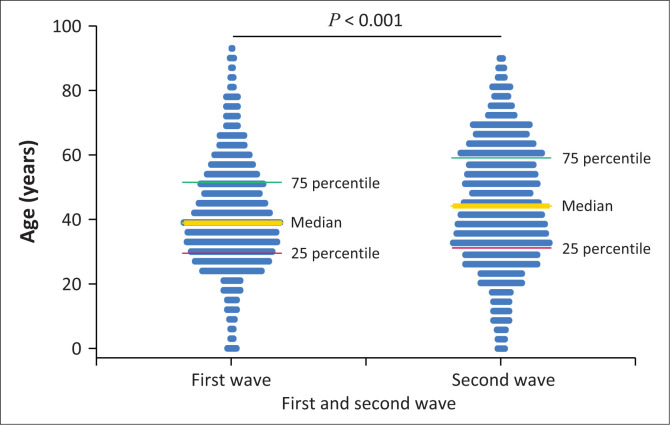
Age analysis of severe acute respiratory syndrome coronavirus-2 real-time polymerase chain reaction results from two major academic hospitals in Gauteng per wave (June 2020 – July 2020 and November 2020 – January 2021).

The result of the Kruskal‒Wallis rank test of age by wave was significant (*p* < 0.001), as shown in Figure 2. The median age was significantly lower in the first wave (39 [30–52] vs. 44 [32–59]) (*p* < 0.001). The sample frequency and percentage per age group in each wave are summarised in Table 2.

**TABLE 2:** Gene target cycle threshold per age group analysis of severe acute respiratory syndrome coronavirus-2 real-time polymerase chain reaction results from two major academic hospitals in Gauteng per wave (June 2020 to July 2020 and November 2020 to January 2021).

Age group (years)	*N*				*E*				*RdRp*				*N*			
	Wave 1		Wave 2		Wave 1		Wave 2		Wave 1		Wave 2		Wave 1		Wave 2	
	*n*	%	*n*	%	Median	IQR	Median	IQR	Median	IQR	Median	IQR	Median	IQR	Median	IQR
0–12	43	3.4	44	3.9	25.0	22.1–27.5	24.65	19.5–28.3	26.1	23.1–29.2	26.6	20.6–32.2	27.8	24.4–32.1	28.8	22.3–33.3
13–18	24	1.9	25	2.2	23.3	17.6–28.5	25.55	21.7–28.6	24.1	19.2–29.4	26.1	23.1–29.0	26.2	20.6–32.6	28.5	25.2–31.7
19–35	409	32.7	296	26.0	23.5	19.4–28.1	25.8	21.3–29.1	25.6	21.4–30.3	27.5	22.9–31.2	27.8	23.1–33.6	29.7	25.2–34.7
36–50	421	33.6	321	28.2	24.2	19.6–28.4	26.9	21.6–30.0	26.3	22.0–30.9	28.7	23.2–31.8	28.2	23.6–33.5	30.5	25.1–34.5
51–70	269	21.5	362	31.8	24.8	21.3–28.2	27.4	23.3–30.8	26.8	22.9–30.5	29.1	25.3–32.7	28.1	24.0–33.1	31.0	26.5–34.8
71 >	65	5.2	86	7.7	23.1	19.3–27.5	24.95	21.5–28.9	25.8	21.7–30.2	26.1	22.5–30.7	27.2	22.4–32.1	28.4	24.6–33.4

*E*, envelope gene; IQR, interquartile range; *N*, nucleocapsid gene; *n*, frequency; *RdRp*, ribonucleic acid-dependent ribonucleic acid polymerase gene.

### Cycle threshold analysis

The results of the Ct trend analysis per wave (independently and within age and sex parameters) of SARS-CoV-2 gene targets (*E, RdRp* and *N*) are summarised in Table 2, Table 3 and Table 4.

**TABLE 3:** Gene target cycle threshold analysis of severe acute respiratory syndrome coronavirus-2 real-time polymerase chain reaction results from two major academic hospitals in Gauteng per wave (June 2020 to July 2020 and November 2020 to January 2021).

Gene target	Wave 1		Wave 2	
	Median Ct	IQR	Median Ct	IQR
*E*	24.1	19.7–28.2	26.4	21.9–30.1
*RdRp*	26.1	23.0–30.4	28.2	23.5–31.8
*N*	27.8	23.4–33.2	30.1	25.3–34.5

Ct, cycle threshold; *E*, envelope gene; IQR, interquartile range; *N*, nucleocapsid gene; *RdRp*, ribonucleic acid-dependent ribonucleic acid polymerase gene.

**TABLE 4:** Gene target cycle threshold per sex cohort analysis of severe acute respiratory syndrome coronavirus-2 real-time polymerase chain reaction results from two major academic hospitals in Gauteng per wave (June 2020 to July 2020 and November 2020 to January 2021).

Categories	Female		Male	
	Median	IQR	Median	IQR
**Wave 1**				
*E*	24.4	20.0–28.6	23.3	23.2–33.2
*RdRp*	26.3	21.9–30.5	25.3	22.0–30.0
*N*	28.2	23.7–33.2	27.2	23.2–33.2
**Wave 2**				
*E*	26.2	21.5–29.9	26.7	22.7–30.5
*RdRp*	28.0	23.1–31.8	28.3	24.3–31.9
*N*	30.1	25.0–34.2	30.1	26.2–34.7

Ct, cycle threshold; *E*, envelope gene; IQR, interquartile range; *N*, nucleocapsid gene; *RdRp*, ribonucleic acid-dependent ribonucleic acid polymerase gene.

### Ct value trends within age groups per wave

The Ct values of each SARS-CoV-2 gene target within age groups per wave were determined and are summarised in Table 4. Overall, non-significant differences were detected when analysing differences in the *N* gene (as is the most reliably detected) among the age groups per wave. The age groups 13–18 years and 71 > years had the lowest median *N* gene Ct value in the first and second wave respectively (26.2 and 28.4).

The first wave had a higher proportion of tests performed in the younger age groups of 19–35 years and 36–50 years while the second wave saw increased testing from the elderly groups (51–70 years and > 71 years).

Table 3 depicts the gene target Ct value trends within and between waves. Significant differences in the median Ct per gene target within and between waves were observed (*p* < 0.001). Median differences in each gene target between waves ranged from 2.0 to 2.3 cycles.

### Ct value trends per sex group

Table 4 depicts the gene target Ct value trends within each sex group per wave. The differences observed were significant at *p* < 0.001. Overall, men had lower median Ct values in the first wave (differences ranging from 1 to 1.1 cycles) with this trend reversed in the second: women had mostly lower median Ct values per target with differences ranging from 0.3 to 0.5 cycles.

## Discussion

We found that younger women were more likely to have positive SARS-CoV-2 PCR tests in the first wave, and older men were more likely to have positive tests in the second wave. The Ct values reported in our study differed from international data, while the demographic analysis was consistent with national trends.

Our median Ct value across all gene targets was lower in the first wave than in the second wave. These values likely represent the SARS-CoV-2 gene target Ct value trends of the Wuhan strain, while the Ct value analysis of the second wave may represent the beta variant.^18^ Various pre-analytical factors also influence the Ct values; therefore, these values may represent both the virological and public health Zeitgeist of the study period. Criteria for laboratory testing remained consistent throughout the first two waves leading to large volumes of inpatient and outpatient samples from varied stages of COVID-19 disease. Additionally, samples obtained from community screening and testing initiatives which aimed to mitigate spread of the virus contributed to laboratory workload. Patient samples with extremes of COVID-19 disease stages together with different SARS-CoV-2 variants between waves impact results were generated at bench level.

Nevertheless, these findings are contrary to previous studies describing the viral kinetics of the Wuhan strain and its variants, and subsequently their expected behaviour on RT‒PCR platforms. A French study that analysed 88 375 Ct values demonstrated that SARS-CoV-2 variants return significantly lower Ct values; the results from hospital samples tested using a variant-specific RT‒PCR assay computed a significantly greater Ct value than did the general, non-hospital population, thereby reflecting a more mature phase of the disease process.^19^

South Africa’s first wave was dominated by the Wuhan-Hu-1 strain, while the beta variant was the principal driver of the second wave. The beta variant was significantly more transmissible because of mutations in the receptor-binding domain (K417N, E484K, N501Y), giving greater viral loads and likely lower Ct values in several studies.^20,21,22^ Therefore, the molecular profile of the beta variant may translate to relatively lower Ct values.

In contrast, we observed higher Ct during the beta wave with multiple virological and non-virological variables accounting for this finding.

Pre-analytical factors may better justify our study’s contrarian Ct findings. In the first wave, patients presented earlier to healthcare facilities than during the second wave, which may have given higher viral loads and lower Ct values during the first wave.^23^ Another pre-analytical factor includes the reduced patient and sample burden of the first wave compared to the second wave, with more prompt testing and processing at earlier clinical presentations.^24^ Globally, the second wave saw higher testing volumes, with healthcare systems in developing countries pushed to the brink of collapse.^25,26^

As COVID-19 progresses, viral titre in the upper respiratory tract gradually declines as symptoms resolve. Because of the second wave’s catastrophic impact on healthcare infrastructure, both patients seeking medical attention and their samples were delayed in being processed.^27^ Combined with limited refrigerated storage facilities, this delay may have deleteriously impacted on the integrity of viral nucleic acid present in clinical samples and may have led to higher Ct values.^28,29,30^.

Therefore, using Ct values as indicators of SARS-CoV-2 viral load or infectivity is unreliable; analytically these methods often show Ct variability of the same strain, variant or even individual samples between manufacturers.^31^ Inconsistencies in laboratory practices, such as varied sample handling and storage protocols, using different extraction and amplification platforms and the implementation of in-house assays, impede the accurate comparison of Ct values within and between centralised laboratories.^32^ Additionally, various chemistries, probes and primer designs prohibit the direct comparison of Ct values generated from different kits.

While the differences observed in our study may not impact on the qualitative detection at higher viral loads, detection at lower titres may be impacted especially if laboratories define positivity at cycles below manufacturer cut-offs.

Hence, Ct values should be used cautiously as indicators or predictors of disease severity and recovery or as assessments of infectivity. Interpretation of the clinical and epidemiological context with laboratory findings should guide patient management and public health policy.

The American Chemical Society has determined that that infectivity may correlate with a Ct below 34; however, the authors suggest that each laboratory determines its own infectivity-Ct correlation owing to variations in testing protocols.^33^

We found that most median Ct values per SARS-CoV-2 gene target across age groups in both waves were below 30, suggesting shedding of viable virus and the likelihood of infectivity across age groups.^34,35^ The magnitude of Ct values among the age groups in both waves was similar and unlikely to alter the qualitative interpretation of the results and suggesting similar (albeit not identical) viral load ranges. This observation is in keeping with international findings. A retrospective study from South India that analysed 5563 hospital results demonstrated significantly lower Ct values among age groups, with differences unlikely to modify the interpretation of the results.^36^

The study’s results emphasise the significance of children as vectors for the transmission of the virus, given their tendency to be asymptomatic or exhibit milder symptoms within the first and second waves, and possibly throughout the pandemic.^24^ However, a recent meta-analysis of 53 studies concluded that although the overall pattern of infection in the paediatric and adolescent groups shared significant homology with earlier lineages, the risk of transmission increased with age.^37^

We found that in both waves, more women were tested than men, which may be explained by women being more likely to engage with healthcare services and being at greater risk of infection.^38,39,40^ Various social determinants and the disproportionate role of women in carer and frontline worker positions could contribute to increased SARS-CoV-2 incidence.^41^ Compared with Black African men, South African Black and Mixed race women infected with SARS-CoV-2 had increased rates of admission but not mortality risk.^42^

Studies conducted in China that included inpatient cohorts suggested equal infection risk among the sexes,^12^ while a modelling study based on German population dynamics suggested an increased risk of infection among women of working age.^43^ In a large cohort of hospital-based patients in the United States of America, men had higher positivity rates than women.^44^ Our findings are consistent with South Africa’s SARS-CoV-2 epidemiological trends, which also demonstrated a greater incidence in females.

In South Africa, the second COVID-19 wave was associated with increased hospital admissions overall, with older people (> 40 years) at increased risk for complicated disease and mortality.^42^ Nationally, the laboratory-confirmed SARS-CoV-2 case burden was dominated by the 30–34 years and 35–39 years age groups in both waves.^31^ Our study supported these national trends,^42^ as the median age of patients who tested positive at the two hospitals was lower in the first wave than in the second. Most testing in the first wave was performed among economically active young adults, while the second wave saw an increase in older cohorts, as this group was at elevated risk of severe disease and therefore more likely to present to a hospital for testing and management.

National testing data depicting the dominant age groups of laboratory-confirmed cases do not discriminate between hospital or non-hospital cases. Disease epidemiology and pathogenesis may cause older outpatients to present to hospitals because of complicated disease compared to younger cohorts, as seen in our study. Globally, age has remained a major risk factor for severe COVID-19^45^; in South Africa, the second wave saw increased mortality compared to the first, especially in the elderly population.^46^

Age was analysed by sex in each wave, and the median age of women was lower than men in both waves, indicating that the men who presented for testing were older. This is synonymous with local epidemiology, as older men have increased risk for complicated disease and are therefore more likely to present to hospital units for specialised care.^42^ Our findings concur with various international studies, such as multinational health database analyses, which described the age by sex of patients tested for SARS-CoV-2, confirming that older men who tested positive were more likely to be hospitalised.^47^

### Limitations

Our study had several limitations. The smaller sample size of the second wave in our study alludes to the increased use of other kits and changes in public health and institutional testing policies. Various pre-analytical and analytical confounders may impact our findings, such as the health-seeking behaviours and biases in the female cohort, which generally result in an earlier presentation to health settings. Different testing conditions and interpretations of guidelines affected sampling techniques and practices among healthcare workers, impacting assay performance. The effect of body mass index (BMI) in males and females on SARS-CoV-2 viral load were not accounted for in our study as BMI was not analysed.^48^

The analytical factors that may impact our findings include the Seegene Allplex 2019-nCoV™ kit’s possibly delayed amplification of targets when testing variants of SARS-CoV-2. The unavailability of SARS-CoV-2 viral load platforms or standard curves (data suggest that Ct values < 20 translate to higher viral loads) impedes accurate assessment of viral titres. Each wave was dominated by a unique variant. Hyperlocal genomic data depicting the circulating variants that drove each wave within Tshwane are lacking, and the subsequent effect of these variants on the Seegene Allplex 2019-nCoV™ requires further study. The study facility is a quaternary unit with a varied patient profile including inpatients and outpatients with both screening and diagnosis of SARS-CoV-2 performed. Therefore, the differences in viral kinetics in these sub-populations may also impact our findings as these details were not specified in our dataset.

## Conclusion

Our study provided granular insight into SARS-CoV-2 epidemiology and cycle threshold value trends during the first two COVID-19 waves in South Africa. The patient demographic analysis agreed with South Africa’s COVID-19 epidemiological findings in both waves. Our study showed that patients tested in the first wave had lower Ct values than those tested in the second wave. All age groups in both waves demonstrated significant infectivity potential. Further studies examining the association between Ct and hospitalisation using genomic data are warranted and may provide further insight into the molecular characteristics and clinical impact of viral variants during the first two COVID-19 waves in South Africa. Also, analysis focussing on the impact of pre-analytical delays in sample processing may shed light on these data.
